# Resource Crafting: Is It Really ‘Resource’ Crafting—Or Just Crafting?

**DOI:** 10.3389/fpsyg.2019.00614

**Published:** 2019-03-20

**Authors:** Qiao Hu, Wilmar B. Schaufeli, Toon W. Taris, Akihito Shimazu, Maureen F. Dollard

**Affiliations:** ^1^School of Management, Zhejiang University of Technology, Hangzhou, China; ^2^Department of Social, Health and Organisational Psychology, Utrecht University, Utrecht, Netherlands; ^3^Department of Work, Organisational and Personnel Psychology, KU Leuven, Leuven, Belgium; ^4^Center for Human and Social Sciences, College of Liberal Arts and Sciences, Kitasato University, Tokyo, Japan; ^5^Centre for Applied Psychological Research, University of South Australia, Adelaide, SA, Australia

**Keywords:** job-related resources, job crafting, individual and team, work engagement, COR theory

## Abstract

This study aims to provide an integrated perspective on job crafting and its antecedents through the exploration of the joint effects of individual-level and team-level job crafting on employee work engagement. Drawing on conservation of resources (COR) theory, we propose that engaging in job crafting behaviors is promoted by the presence of job-related resources. In turn, job crafting is expected to result in higher levels of work engagement. We expect this reasoning to hold for the individual as well as the team/collective levels. The hypotheses were tested using data from 287 medical professionals from 21 hospital units of a Chinese public hospital. Findings from two-level Bayesian structural equation modeling supported the idea that at the individual level, individual job crafting behaviors partially mediated the relationship from individual resources to individual work engagement. Further, collective crafting mediated the relationship from team resources to individual work engagement. In addition, a positive cross-level relation between collective crafting and individual crafting was found. We conclude that stimulated by resources, both job crafting processes at the individual-level and team-level can promote individual work engagement in Chinese employees.

## Introduction

Job crafting is a means by which employees can cultivate positive meaning in their job (Wrzesniewski et al., 2013). A positive outcome of this is work engagement defined as “a positive psychological state characterized by energy investment and psychological presence” (Sonnentag, 2017, p. 14). The antecedents of job crafting have largely been discussed by scholars in terms of individual level factors such as perceived job-related characteristics, such as job resources, as well as individual workers’ needs, goals, and behaviors (Bakker et al., 2012b; Tims et al., 2012; Van den Heuvel et al., 2015). However, these antecedents usually occur in an *organizational* context, suggesting that social and team-level factors may also be relevant in shaping the degree to which individuals engage in job crafting behaviors and the outcomes thereof (e.g., Leana et al., 2009; Hu et al., 2016). That is, people are not always motivated by personal concerns; instead, individual motivation is also reflected in, informed by, and adapted to the needs, goals, or expectations of other team members (Ellemers et al., 2004; Bizzi, 2016).

As we will argue a more comprehensive multilevel view of how resources spur crafting and relate to engagement is needed. In explanation of how and why job resources influence job crafting we draw on conservation of resources (COR) theory (Hobfoll, 1989, 2011) which assumes that much of human behavior is organized around the acquisition and preservation of valued resources – resources are required to directly or indirectly fuel psychological energy needed for surviving and thriving. Research has revealed that job-related resources (i.e., the features of the job that produce benefits that satisfy one’s specific needs, such as the opportunities for learning and development, conditional resources such as good remuneration policies, and personal resources such as high professional efficacy) are key factors in maintaining psychological energy and motivation at work (Xanthopoulou et al., 2007; Taris et al., 2010; Hu and Schaufeli, 2011; Hu et al., 2017c) and can stimulate job crafting (Tims et al., 2014; Heuvel et al., 2015). Beyond these individual processes, we propose team-based CORs view in explanation of processes at the team level that link job resources to job crafting and in turn engagement. Building on COR theory (Hobfoll, 1989), we seek to better explain and understand how job-related resources lead employees toward more optimal functioning in individual and team contexts.

### COR Theory and Job Crafting

Conservation of resources (COR) theory assumes that resources have an intrinsic motivational element that facilitates the attainment of goals and the satisfaction of needs (Van den Broeck et al., 2008). Individuals strive to invest, foster, and protect those centrally valued resources with the expectation of receiving positive outcomes in return (Hobfoll, 1989, 2011). These resources refer to a broad psychological concept that is not restricted to job-related factors, and also includes universal psychological resources such as meaningfulness and need satisfaction (Deci and Ryan, 2000; Wrzesniewski and Dutton, 2001). Those universal psychological resources play an important role in individuals’ thriving, while thwarting of those resources have an energy-depleting effect (Deci and Ryan, 2000; Hobfoll, 2011). COR further stresses that it is not necessarily the workers with abundant resources who thrive but rather the workers who are best able to allocate those resources to maximize their resources reservoirs (Halbesleben et al., 2014). This means that individuals with a pool of job-related resources are better able to invest and gain additional resources. Note that these additional resources are not limited to job-related resources, but may also include universal psychological energy resources.

*Job crafting* is the self-initiated behavior that workers use to proactively shape work practices so that these align with their own personal preferences, values and skills (Wrzesniewski and Dutton, 2001; Slemp and Vella-Brodrick, 2013). Job crafting is important for individuals in the process of maintaining and supplementing psychological energy resources, because job crafting facilitates the experience of meaningfulness and work identity (Wrzesniewski et al., 2013). Viewing one’s own job as more purposeful and meaningful can produce significantly more psychological energy. People holding many resources are motivated to put more effort into their crafting actions to shape their work into a meaningful job (Wrzesniewski and Dutton, 2001; Malo et al., 2016), e.g., by incorporating new tasks, reducing hindering tasks, or deepening their social bonds at work (Tims et al., 2012; Niessen et al., 2016). Research has revealed that meaningfulness is an energy resource that promotes work engagement, even when job resources are controlled for (May et al., 2004; Byrne et al., 2016). By capturing positive work meaning, job crafting can improve momentary psychological energy resources and boost work motivation. In turn, psychological energy has the potential to boost work engagement. Based on this idea, it is possible to consider job crafting from the within the context of job-related resources and work engagement.

### Job-Related Resources and Motivations at Individual-Level and Team-Level

Job-related resources is a broad umbrella concept that comprises various kinds of objects and conditions referring to one’s work. Two forms of job-related resources are distinguished at the individual level and at the team level, depending on whether they are based on collective attributes (or societal identities) or on individual characteristics. *Individual-level resources* can be conceptualized as “energy reservoirs” at work that individuals can tap into to regulate their demands (Hobfoll, 1989, 2002), such as professional efficacy (a personal resource; Xanthopoulou et al., 2007), opportunities for development (i.e., an energy resource; Hu et al., 2016), and remuneration (i.e., a conditional resource; Hu and Schaufeli, 2011). Such resources are directly related to person-specific requirements, thereby meeting the needs of and taking on meaning and value for individual workers. Individual resources, to some degree, reflect individual identities in the workplace, and richer individual resources represent better individual identities. People will strive to preserve a given level of individual resources in order to protect and maintain their identities. These individual resources have a motivational element to them that promotes individuals to engage in self-regulatory behaviors that help them to protect or acquire psychological energy.

Team identity requires that team membership is assimilated into the self-concept of the individual team members, i.e., they should consider themselves to be part of the collective (Ellemers et al., 2004). The identification with a team originates from the reciprocal relationships team members maintain, involving shared norms and mutually beneficial interactions (Thomson and Perry, 2006). As such, team members partly define themselves in terms of a social referent (i.e., the other team members) and they tend to develop similar perceptions of how their team is cooperative or works effectively. If so, this creates a situation in which team-based (or collective) needs, expectations, and goals are regarded as *relative* intrinsic sources of motivation (Ellemers et al., 2004). *Team-level resources* are endorsed with shared values, serve the interests of a team, coordinate team members’ actions toward team goals and, hence, they are regarded as motivational sources (Edwards and Cable, 2009). For example, team effectiveness during a field study program among nurses conducted over an 8-week period led team members to adapt to changing environments (Gibson, 1999). Furthermore, team cooperation helps team members to recognize physiological and emotional cues that can create challenges in meeting work goals; and team learning increases the accuracy and frequency of communication among team members, providing them with access to a broader range of alternative perspectives (Johnson and Johnson, 1999; Carpenter et al., 2004). Note that when situational features encourage people to regard their self-identities in collective terms, they are not necessarily driven by personal considerations only. Alternatively, if team identity is internalized as part of a person’s sense of self, they establish goals for the team and respond to team goal achievement in essentially the same manner as they respond to achieving their own goals (Shaw, 1981). The motivation to attain a collective outcome is regarded as originating from individual concerns and motives (Ilgen and Sheppard, 2001; Ellemers et al., 2004).

### Individual Crafting, Collective Crafting and Individual Work Engagement

*Individual crafting* may be defined as the self-initiated changes employees make to their job in order to optimize their functioning in terms of well-being, attitudes or behavior (Vanbelle et al., 2013). Individual crafting may encourage self-regulation strategies that maximize individual-level outcomes. If employee motives, strengths, and passions tap into valued personal needs and abilities, they are likely to engage voluntarily in activities they consider important and of which they are capable. A review on the correlates of work engagement revealed that individual crafting is a crucial factor that helps staying engaged at work (Bakker et al., 2012a). Petrou et al. (2012) found that individual crafting by seeking challenges and reducing demands was positively associated with day-level work engagement. Similarly, Tims et al. (2013) found that individual crafting had a positive impact on employee work engagement through introducing changes in their job demands and job resources. Apparently, individual crafting is a means to customize individual’s valued psychological energy resources in ways that optimize situational features as well as worker well-being and functioning.

People place the greatest importance regarding their self-definitions on aspects of their identity that best satisfy their particular motives (Easterbrook and Vignoles, 2012). This *social identity* can help define the circumstances under which people are likely to consider themselves as either independent individuals or as part of a collective. When circumstances foster a conception of the self in individual terms, individual considerations are key drivers of work motivation (Ellemers et al., 2004). Conversely, when individuals conceive of themselves in collective terms, this self-conception energizes people to exert effort on behalf of the team, facilitating them to direct their effort toward attaining collective goals.

*Collective crafting* (also called collaborative or shared crafting) refers to the activities carried out by team members with a collective cognition to jointly change the nature of work practices and processes (Leana et al., 2009; McClelland et al., 2014; Makikangas et al., 2016). Collective crafting is executed in a reciprocal fashion. Workers tend to engage in collective crafting, depending on the extent to which these seem to be individually rewarding. Collective crafting involves forging commonalities from individual differences, with a focus on the individual’s meaning of work aligning with team identity. The congruent expectations of self-interests and collective interests lead employees to develop a collective self-concept, energizing them to exert effort on behalf of their team in order to achieve collective goals and outcomes. These mutually beneficial relationships, the development of trust and modes of reciprocity can satisfy individual psychological needs and energies as well. The acquisition of psychological energy in collective crafting is also likely to encourage employees into a course of action and continue or expand their work engagement. For example, the study of Mäkikangas et al. (2017) showed that self-efficacy for teamwork was positively associated with team job crafting behavior at the individual level, and team features (team cohesion and climate) were positively related to daily team job crafting at both the within- and between-team levels. Tims et al. (2013) found team job crafting related positively to individual performance via individual job crafting and individual work engagement.

In sum, individual resources and team resources have motivational elements that facilitate the attainment of goals and the satisfaction of needs. As such, individual resources and team resources act on psychological energy to drive employee behaviors. Individual resources encourage an individual-level conception, and this will lead people to engage in individual crafting. Similarly, team resources encourage a conception of the self in collective terms, and this will stimulate people to exert collaborative efforts into collective crafting. Both individual crafting and collective crafting are central in allowing people to choose and most fully develop preferred ways of work to optimize their psychological energy. This will bring about more purposeful and meaningful work, which will in turn lead to higher work engagement. As a result, individual work engagement will not only be affected by individual crafting, but also by collective crafting. Based on these notions, two hypotheses are proposed:

*Hypothesis 1.* Individual resources are positively related to individual work engagement through individual crafting.

*Hypothesis 2.* Team resources are positively related to individual work engagement through collective crafting.

## Materials and Methods

### Sample and Procedure

The study was conducted in March 2016 as part of a collaborative research project that primarily focused on employee well-being in a public hospital of a Chinese university. The hospital included 21 medical units and 408 registered medical employees. Prior to the study, permission to conduct this study was obtained from the hospital head and the survey content was discussed with a hospital administrative who was responsible for this project. 350 paper-and-pencil questionnaires were distributed by the researchers among all medical professionals who were present during a regular monthly staff meeting. Participants were encouraged to fill in the questionnaire and confidentiality was assured. In total, 287 valid questionnaires were returned (82% response rate), including 59 doctors, 161 nurses, and 67 medical technicians. Of the participants, 222 were female (77.4%) and 56 were male (19.5%), 9 participants did not provide gender information (3.1%); their mean age was 31.44 years (*SD* = 8.48), and their average tenure was 9.00 years (*SD* = 8.10).

### Measures

*Individual job crafting* was measured using the Overarching Job Crafting Scale (O-JCS, Vanbelle et al., 2013; Hu et al., 2017a,b). The O-JCS focuses on the changes employees make in their jobs to optimize their functioning in terms of well-being, work-related attitudes and behavior (cf. Vanbelle et al., 2014). The four items are “I make changes in my job to feel better,” “I change my job so it fits better with who I am,” “I make changes in my job to perform better,” and “I change my job so it fits better with what I think is important” (1 = “strongly disagree,” 5 = “strongly agree”). Cronbach’s alpha for this measure was 0.93. Tests showed that raters from the same team had a high level of within-group agreement [*r*_wg(j)_ = 0.83], the intraclass correlation coefficients [ICC(1) = 0.14], and the reliability of the mean [ICC(2) = 0.68].

*Collective crafting* was measured using three items of the Collaborative Crafting scales (McClelland et al., 2014). These were “In the past 12 weeks (without supervisory/management input) to what extent has your team …” (1) “changed the approach it uses to make the work more interesting”; (2) “adjusted the tasks it undertakes to make the job more fulfilling”; and (3) “changed the variety of work tasks it performs to make the work more meaningful?” Since collective crafting practices are embedded within social networks, trust promotes efficient cooperation among interdependent actors (McAllister, 1995). We therefore added an item to capture relational crafting: (4) “changed interpersonal relationships at work to increase mutual trust.” All items employed a 5-point scale ranging from 1 (“not at all”) to 5 (“a great deal”). Cronbach’s alpha for this measure was 0.96. Because of the high level of agreement between raters within the same team [*r*_wg(j)_ = 0.81, ICC(1) = 0.13, ICC(2) = 0.66], we averaged the responses of employees within each team to create an aggregated measure of team-level collective crafting.

*Individual-level resources* were assessed with subscales of the Questionnaire on the Experience and Evaluation of Work (QEEW; Van Veldhoven et al., 2002). Three individual-level resources were included. The first was *remuneration* (four items, e.g., “Can you live comfortably on your pay?” cf. Hu and Schaufeli, 2011), with a Cronbach’s alpha of 0.94. Tests showed that raters from the same team had an adequate agreement regarding individual crafting [*r*_wg(j)_ = 0.76, ICC(1) = 0.07, ICC(2) = 0.49]. The second was *opportunities for learning and development* (four items; e.g., “In my job I have the possibilities to develop my strong points”; see also Hu et al., 2011), Cronbach’s alpha is 0.85, the *r*_wg(j)_ is.76, the ICC(1) is 0.08 and the ICC(2) is 0.53. The third was *professional efficacy* (five items, e.g., “When difficult problems happen at work, I know how to solve them.”). Cronbach’s alpha is 0.92, the *r*_wg(j)_ is 0.84, the ICC(1) is 0.14 and the ICC(2) is 0.68. All items were on a 5-point Likert-type scale ranging from 1 (“never”) to 5 (“always”).

*Team-level resources* were assessed by the two subscales of Liu’s (2009) Shared Leadership Scale: (1) *team cooperation* (3 items, e.g., “My team members cooperate in each other’s work,” Cronbach’s alpha was 0.94); (2) *team learning* (3 items, e.g., “My team improves professional capabilities by brainstorm and seminar,” Cronbach’s alpha is 0.93); and one self-constructed scale *team effectiveness* (3 items, namely “Does your hospital unit cooperate effectively?”, “Are the working arrangements in your hospital unit properly fulfilled?”, and “Does everyone in your hospital unit work to the best of his or her abilities?”, Cronbach’s alpha is 0.87). All items were answered on a 5-point Likert-type scale ranging from 1 (“never”) to 5 (“always”). Tests revealed that the raters from each team had an adequate agreement regarding team-level job resources: team cooperation [*r*_wg(j)_ = 0.73, ICC(1) = 0.20, ICC(2) = 0.76], team learning [*r*_wg(j)_ = 0.74, ICC(1) = 0.09, ICC(2) = 0.56], and team effectiveness [*r*_wg(j)_ = 0.83, ICC(1) = 0.21, ICC(2) = 0.78]. We, therefore, averaged the responses of the employees within each team to create aggregated measures of team-level team cooperation, team learning, and team effectiveness.

*Work engagement* was assessed with the 9-item short version of the Utrecht Work Engagement *Scale-9* (*UWES-9;* Schaufeli et al., 2006). The three components of engagement were measured with three items each: *vigor* (e.g., “At my work, I feel bursting with energy”), *dedication* (e.g., “My job inspires me”), and *absorption* (e.g., “I get carried away when I am working”), with a response scale 1 = “never” and 5 = “always” (cf. Li et al., 2013; Mao and Tan, 2013; Sun et al., 2015). Cronbach’s alphas were 0.86, 0.90, and 0.91, respectively. Tests revealed that the raters from same team had an adequate agreement regarding vigor [*r*_wg(j)_ = 0.75, ICC(1) = 0.17, ICC(2) = 0.82], dedication [*r*_wg(j)_ = 0.74, ICC(1) = 0.19, ICC(2) = 0.70], absorption [*r*_wg(j)_ = 0.75, ICC(1) = 0.20, ICC(2) = 0.76].

### Data Analysis

The median percentage of the studied variables with a missing outcome was 1.4% (range from 0.3 to 2.1%). Missing data were handled with series mean values substitution. Level-1 variables were group-mean centered, while Level-2 variables were grand-mean centered. As the number of level-2 observations (i.e., the 21 hospital units) in this study is relatively small, a two-level Bayesian structural equation modeling (BSEM) as implemented in Mplus 7 (Muthén and Asparouhov, 2012; Muthén and Muthén, 2015) was used to test the hypothesized model. Compared to Maximum likelihood estimation, Bayesian estimation methods are more reliable in small samples and can handle complex models better (Muthén and Asparouhov, 2012; Hox et al., 2014).

Prior to estimating the hypothesized model, a single-level exploratory factor analysis was conducted to check whether the individual-level and team-level job resources could empirically be distinguished from each other. Exploratory factor analysis is primarily a data-driven approach, and few restrictions are placed on the patterns of relations between the common factors and the measured variables (Fabrigar et al., 1999). In the absence of strong evidence-based expectations for the distinction between individual-level and team-level resources, we decided that this exploratory approach was more suitable than a confirmatory analysis.

Given the potential convergence problems of small sample sizes [i.e., the team-level sample size was 21, and the individual-level sample sizes ranged from 5 to 25 participants (the average number of participants was 13)], we simplified the measurement part of the BSEM model by aggregating the team-level scores across employees from the same team and by using three latent regression factor scores for the variables studied (i.e., individual-level resources, team-level resources and work engagement).

**FIGURE 1:**
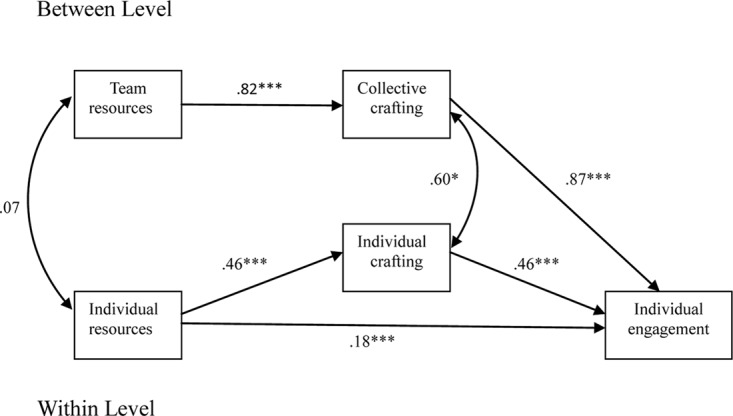
Path diagram of the final model. ^∗^*p* < 0.05, ^∗∗^*p* < 0.001.

To estimate the hypothesized two-level model, four nested Bayesian SEM models were compared: (a) a null model (Model 0), where all structural paths among the five concepts were assumed to be zero. This model presents a baseline model against which the other models can be compared; (b) a within-level model (Model 1) with the effects of the explanatory variables at the individual-level and the structural paths at the team-level fixed at zero. Model 1 allowed us to assess the effects of individual resources on individual work engagement, which were further mediated by individual job crafting; (c) a full two-level model (Model 2) in which the structural paths at the team-level were released to estimate the effects of team-level resources on team-level crafting; and (d) a cross-level model (Model 3, see Figure 1) that states that team-level crafting influences individual work engagement. The model includes mediation in cross-level design, in which within-level variables are assessed at the between-level (Preacher et al., 2010). In addition, two cross-level covariances between team resources and individual resources, and between collective crafting and individual crafting were included (denoted with double-headed arrows in Figure 1). The first three steps (M0–M2) provide information as to whether multilevel analysis is justified. The final step (M3) tests the hypothesized relationships in this study.

Posterior predictive checking was used to detect model misspecifications. A low posterior predictive *p*-value (PP*p*) and a positive lower limit of the 95% confidence interval for the difference for the real and replicated data (95% PP*p* interval) indicate poor fit (Muthén and Asparouhov, 2012). There is no particular cutoff value available that signifies whether a particular PP*p* value indicates that a particular model should be considered as ill-fitting (Muthén and Asparouhov, 2012). However, Muthén and colleagues commonly use a PP*p*-value of.05 in their simulation studies (Asparouhov and Muthén, 2010; Muthén and Asparouhov, 2012). Further, when comparing two or more nested models, the model with the lowest DIC (Deviance Information Criterion) has the best fit (Gelman et al., 2004).

## Results

### Preliminary Analyses

We conducted a single-level preliminary analysis. The means, the standard deviations, and the correlations for the study variables are displayed in Table 1. A principal components factor analysis with varimax rotation for the six resource variables was conducted. Table 1 shows that team cooperation, team learning and team effectiveness loaded on a single team resources component (eigenvalue = 2.84), whereas learning and development opportunities, remuneration, and professional efficacy loaded on an individual resources component (eigenvalue = 1.77). Hence, the pattern of loadings of the indicators of the two kinds of resources showed that participants distinguished between individual-level and team-level resources.

**Table 1:** Factor loadings of the resource indicators (*N* = 287).

	Component 1	Component 2
Team resources:		
Team learning	0.93	0.14
Team effectiveness	0.93	0.09
Team cooperation	0.91	0.05

Individual resources:		
Remuneration	0.06	0.83
Professional efficacy	0.09	0.83
Development opportunities	0.01	0.78

Explained variance	43.16%	33.75%
Initial Eigenvalues	2.84	1.77


The means, standard deviations, and correlations of within-individual estimates and between-team estimates of the focal variables are presented in Table 2. Bivariate within-individual variables correlations showed that individual crafting was positively related to the indicators of individual resources (*r* ≥ 0.33, *p* < 0.01) and the three indicators of work engagement (*r* ≥ 0.54, *p* < 0.01). Bivariate between-team variables correlations showed that collective crafting was positively related to the indicators of team resources within-individual estimates (*r* ≥ 0.80, *p* < 0.01).

**Table 2:** Description of sample and correlations (single-level analysis, *N* = 287).

Variable	*M*	*SD*	1	2	3	4	5	6	7	8	9	10
(1) Remuneration	3.20	0.77	–									
(2) Development opportunities	3.19	0.85	0.47^∗∗^	–								
(3) Professional efficacy	3.52	0.68	0.58^∗∗^	0.47^∗∗^	–							
(4) Individual crafting	3.20	0.71	0.33^∗∗^	0.49^∗∗^	0.37^∗∗^	–						
(5) Vigor	2.85	0.99	0.32^∗∗^	0.45^∗∗^	0.20^∗∗^	0.54^∗∗^	–					
(6) Absorption	2.67	0.98	0.30^∗∗^	0.46^∗∗^	0.18^∗∗^	0.57^∗∗^	0.82^∗∗^	–				
(7) Dedication	2.70	1.01	0.37^∗∗^	0.49^∗∗^	0.23^∗∗^	0.56^∗∗^	0.88^∗∗^	0.86^∗∗^	–			

(8) Collective Crafting	3.16	0.36	–	–	–	–	–	–	–	–		
(9) Team effectiveness	3.69	0.33	–	–	–	–	–	–	–	0.88^∗∗^	–	
(10) Team cooperation	4.00	0.36	–	–	–	–	–	–	–	0.82^∗∗^	0.76^∗∗^	–
(11) Team learning	3.68	0.29	–	–	–	–	–	–	–	0.80^∗∗^	0.85^∗∗^	0.78^∗∗^


*The aggregated correlations of team-level variables are presented in the right column. ^∗∗^*p* < 0.01.*

### Model Estimation and Assessment of Fit

Models M0–M3 are nested, in that each subsequent model released additional regression paths or (co)variances as compared to the previous, simpler model. All paths at the individual level showed positive relations in the within-level model (M1). The full model with explanatory variables in the two-level model M2 showed adequate fit (PP*p* = 0.17; see Table 3). All regression paths in this model were significant, at both the within and the between levels.

**Table 3:** Regression coefficients and model fit information of the nested two-level BSEM models.

	Estimate (Posterior *SD*)			
	Model 0	Model 1	Model 2	Model 3
Level 1 (individual-level, *N* = 287)				
Individual resources →Job crafting	0.00	0.47^∗∗∗^ (0.05)	0.47^∗∗∗^ (0.05)	0.46^∗∗∗^ (0.05)
Individual resources → Work engagement	0.00	0.20^∗∗∗^ (0.06)	0.19^∗∗∗^ (0.06)	0.18^∗∗∗^ (0.07)
Individual crafting → Work engagement	0.00	0.20^∗∗∗^ (0.06)	0.46^∗∗∗^ (0.06)	0.46^∗∗∗^ (0.06)

*Level* 2 (team-level, *N* = 21)				
Team resources → Collective crafting	0.00	0.00	0.86^∗∗∗^ (0.06)	0.82^∗∗∗^ (0.10)

*Cross-level*				
Collective crafting → Work engagement	0.00	0.00	0.00	0.87^∗∗∗^ (0.14)
Team resources ↔ Individual resources	0.00	0.00	0.00	0.07 (0.31)
Collective crafting ↔ Individual crafting	0.00	0.00	0.00	0.60^∗^ (0.23)

PP*p*	0.00	0.00	0.17	0.27
95% PP *p* interval	[180.33, 231.37]	[16.43, 62.78]	[-11.74, 28.70]	[-16.65, 29.42]
DIC (pD)	2259.54 (47.60)	2085.15 (46.92)	2060.84 (48.93)	2012.05 (44.72)


*PPp, Posterior predictive p-value; DIC, deviance information criterion; pD, estimated number of parameters; ^∗^p < 0.05, ^∗∗∗^p < 0.001.*

Next, the cross-level hypothesized model (M3) was tested. The results revealed that this model fitted the data better than model 2 (PP*p* = 0.27, ΔDIC = -48.79). The final model is shown in Figure 1. At the *individual level*, individual resources were positively related to job crafting and work engagement (βs were 0.46 and 0.46, respectively, *p*s < 0.01), while job crafting related positively to individual work engagement (β = 0.18, *p* < 0.01). At the *team level*, collective crafting related positively to team resources and individual work engagement (βs were 0.82 and 0.87, respectively, *p’*s < 0.01). In addition, a significant cross-level correlation between collective crafting and individual crafting was found (*r* = 0.60, *p* = 0.02), suggesting that individual and collective job crafting behaviors are closely interconnected (cf. the double-headed arrow in Figure 1). However, the cross-level correlation between individual resources and team resources was non-significant (*r* = 0.07, *p* = 0.31). Further, the hypothesized mediation effects were confirmed by a significant indirect effect of individual resources via job crafting on work engagement at the individual level (β = 0.20, *p* < 0.001, Posterior *SD* = 0.04; Hypothesis 1 supported), and a significant indirect effect of team resources via collective crafting on work engagement at the team level (β = 0.23, *p* < 0.001, Posterior *SD* = 0.07; Hypothesis 2 supported).

## Discussion

To date, our understanding of how relatively stable job-related resources influence work engagement is relatively limited. Building on COR theory, this study intended to provide an integrated perspective on the relationships among job-related resources, job crafting and work engagement, assuming that relatively stable job-related resources motivate employees to move toward more optimal functioning by the acquisition of psychological energy resources through the process of job crafting. A cross-level study was conducted to investigate the effects of job crafting on both the individual-level and team-level levels in promoting employee work engagement. Our findings revealed that job crafting is as influential at the team level as it has been found to be at the individual level. Moreover, consistent with COR theory, our findings showed that resources are of paramount importance in the work context. Three sets of findings stand out as especially important: (1) individual resources could be distinguished from team resources; (2) individual-level resources promoted employee work engagement via individual job crafting, while team-level resources promoted employee work engagement via collective crafting; and (3) individual job crafting was associated with collective job crafting: both promoted individual work engagement.

### Theoretical Implications

Our study attempted to integrate the concepts of job crafting, job-related resources and work engagement into one overarching framework by recognizing the importance of psychological energy. It provides valuable insights into the relevance of individual-level job crafting and team-level job crafting processes. Five important theoretical implications deserve mentioning.

First, our study showed that individual-level resources could be distinguished from team–level resources, as suggested by two independent components in the factor analysis and their non-significant cross-level correlation. This is important, since both sets of items were measured by asking individual participants about their individual-level job crafting behaviors as well as job crafting behaviors at the level of their work team. The distinction between the two sets of items tells us that the participants clearly distinguished between both types of items. The reason why the participants did so might be that they differentiate between cues that make salient aspects of personal identity and cues that are relevant to their social identity. Individual-level resources represent the individual’s identity that derives from a unique personal attribute (Turner, 1982), while team-level resources represent team identity that is based on internalized group membership (Ellemers et al., 1999). Individual-level resources are directly related to individual-specific requirements that meet the needs of individuals, but that are not necessary for other team members. Conversely, team-level resources are *relative* intrinsic sources of motivation, providing cues of reciprocity that help team members in establishing mutual cooperation and interpersonal trust. Both types of resources have distinct implications for the individual’s behavior and for the functioning of team. Individual-level resources are focused on personal goals, and are likely to motivate self-interested action. Conversely, team-level resources are more focused on multiple, interdependent requirements, and are more likely produce the possibility for individuals to engage in co-action and collective action. For example, professional efficacy is an employee’s belief in their individual capability to perform a specific task (Taris et al., 2010). However, team effectiveness is not simply the sum of the efficacy beliefs of its individual members, since team effectiveness involves complex patterns of interwoven and reciprocal social influences, more so than does individual efficacy (Kozlowski and Ilgen, 2006).

Second, job-related resources have energizing potential in making the goal more easily attainable and induce greater motivation for the pursuit of well-being (Hu et al., 2017c). Our study confirmed that individual resources are positively linked to individual work engagement. Given that job crafting aims to alter perceptions of the meaning of work and, hence, one’s identity at work (Wrzesniewski and Dutton, 2001), we proposed a definition of individual crafting that fits well with the motivation for job crafting and that allows us to draw upon COR theory. Our study revealed that job crafting bridges job-related resources with employee work engagement. This finding agrees with Hobfoll’s (1998) assumption that people will attach value to particular behaviors if they expect these behaviors to lead to a desired state. Job-related resources play a critical role in motivating workers to initiate and persist in particular work behaviors, because they largely depend on their ability to take advantage of their resources at hand. When people who are in an advantageous position (i.e., who have more job-related resources) identify available opportunities to gain more psychological energy, they are motivated to engage in those crafting activities that allow them get more enjoyment and meaning, enhance their work identities, promote their development, and thrive.

Third, individual identity and social identity are two integral aspects of people’s identity, that is, people tend to attach value to the behaviors that seem to be rewarding for their desired identity (Ellemers et al., 2004). This means that individual behavioral preferences can be adapted to be consistent with collective concerns or collaborative considerations (Ellemers et al., 2004). However, previous work on job crafting has primarily examined individual crafting behaviors and how these affect individual behavior and outcomes. Our study is based on the idea that not only individual-level, but also team-level job crafting may have a substantial effect on individual outcomes. That is, both individual and collective job crafting are assumed to be key strategies in the process of resource conservation, and both may promote individual work engagement. Our study showed that individual-level resources, partially mediated by individual crafting, create the conditions in which employees personally engage. This result is consistent with the view of Maslow (1954) that people need both self-expression and self-employment in work. Individual crafting tends to speak to employees’ personal work identities, while work engagement relates to their own needs, abilities, and preferences. People will experience high levels of work engagement when they independently modify aspects of their jobs to improve the fit of the self and job. The mediational effect of individual crafting on the relation between individual resources and work engagement underlines the notion that people’s autonomous functioning and the attainment of wellbeing are indeed connected, and that job crafting plays a pivotal role in this process.

Fourth, our study showed that team-level resources promoted employee work engagement via collective crafting. One explanation is that when team-level resources reach a sufficiently high level, people will internalize and integrate endorsed shared values, norms and rules, conceiving themselves in collective terms. This will lead them to engage in interdependent and goal-directed actions with other team members, thus affirming their identification with the team. If these actions relate to improving work circumstances and well-being (collective crafting), they could lead to higher levels of vigor and energy. Another explanation relates to the team dynamic that initiates and directs psychological energy. When motivation operates at the team level, interpersonal interactions contribute positively to the experienced meaning of work, by means of a process of emotional contagion (Bakker et al., 2006). This enables people to develop a joint interpretation of the work environment as promoting personal growth and development, which, in turn, may result in increased levels of individual work engagement.

Finally, our study revealed that individual crafting correlates positively to collective crafting at the team level. One explanation is that *collective crafting* is a collaborative action through which autonomous individuals see different aspects of a problem, and search for solutions that go beyond their own limited vision of what is possible. People can use each other’s resources and share information to strengthen their own operations and programs, which will facilitate individual crafting. Another explanation draws on aspects of the Chinese collectivist culture (Hofstede, 1991) in which individuals’ work identity is based on the social system. When people seek the satisfaction of having a job that is well-recognized by their social environment, they tend to be concerned about the impact of their own behavior on their fellow team members (Hui and Triandis, 1986). This creates situations in which individual success ultimately depends on the attainment of collective goals. This not only applies to the individual’s action in terms of one’s own interest, but also in terms of the interests of a collectivity. Thus, individual and joint effort are interdependent in affecting crafting behavior. In addition, a collectivist orientation might provide an alternative explanation for not only the positive relations between individual crafting and collective crafting, but also for the simultaneous effects of individual crafting and collective crafting on work engagement. Our study supports the idea that both individual and collective job crafting are central to optimal human functioning.

### Practical Implications

First, our study showed that individual resources and individual crafting are linked with employees’ work engagement. This suggests that in order to improve work engagement, organizations should provide their employees with enriched job descriptions, thus fostering enriched resourceful workplaces to create meaningful work. Our study further showed that informal job crafting appears to be more effective in promoting employee work engagement than having a formal resourceful context provided by the employer. Thus, managers may foster an empowering work environment by allowing employees to craft their jobs and to maximize the effectiveness of their efforts, thus improving employee wellbeing. Additionally, for employees who are concerned about their own wellbeing, job crafting actually increases their motivation and they are therefore more likely to get involved in activities that promote work engagement, thereby shaping their own work enthusiasm.

Second, our study showed that collective crafting is an effective means to improve employees’ psychological well-being. Managers can provide positive reinforcement to those who act on collective crafting by encouraging employees to participate in staff appreciation programs, such as sharing expertise and knowledge specific to their roles, and by responding openly and supportively to employee suggestions, such as introducing new structures and systems where there are increased resources in which collective crafting promotes work engagement. Given that Chinese cultural factors influence individuals’ behavior in a general way, another suggestion to promote work engagement could be to emphasize collectivist work values (prioritizing team goals over personal interests). This could result in a strong identification with the values and goals of the organization, and could thus help to cultivate dedicated and motivated employees.

Third, our study presents a conceptual framework that may stimulate both researchers and managers to recognize that psychological energy is a crucial element for individuals to flourish. We believe that this is important in itself, as it creates a working model for how different strands within the job crafting literature (e.g., focusing on the reduction of imbalances in job demands and resources, Tims et al., 2012; task crafting, relational crafting and cognitive crafting, Slemp and Vella-Brodrick, 2013; change task and relational boundaries and differentiate cognitive and behavioral changes, Weseler and Niessen, 2016; promote meaningfulness at work, Vanbelle et al., 2013; and alter the structure of tasks, Bizzi, 2016) can influence work engagement. Employees base their perception as to whether they have meaningful work on many different sources of information. These sources of information allow researchers and managers to establish a research agenda that identifies how meaningfulness-related concerns may cause crafting behavior and which kinds of approach people may use.

### Limitations and Suggestions for Future Research

One important limitation of the present study is its reliance on self-report measures, which might have caused common method bias. However, there are good reasons why we used self-assessments of job crafting and work engagement. Respondents have easier access to examples of their own crafting behaviors and wellbeing than others have, and can potentially detect differences between their own behaviors and well-being and that of others. Using self-assessments of job crafting and work engagement, respondents are likely to be more motivated to talk about themselves than others. It is also likely to produce more accurate results by avoiding the potential halo effect of others based their assessments upon overall impressions or observe behaviors designed to impress investigators (Ghitulescu, 2012). Moreover, self-report measures are a well-accepted and valid way of measuring of job crafting and work engagement in the literature (e.g., Schaufeli, 2012; Van Wingerden et al., 2017).

A related limitation is that all team-level variables were derived from individual employee-level data. However, the *r*_wg(j)_ and the ICC-values demonstrated reasonable team-level agreement concerning the team variables and their cross-level effects. Still, in future research on the relationship between individual-level or team-level variables, it would be worthwhile to obtain objective measures, such as the monthly salary to measure an individual’s remuneration, the degree to which team objectives were achieved to measure team effectiveness, and the number of seminars or courses taken to measure team learning. Of course, it would also be worthwhile to ask the team leader to evaluate team-level variables.

A second limitation is the generalizability of the findings of the present study. Our data were collected among a relatively specific sample of Chinese medical professionals. Although these professionals worked in a general hospital, it cannot be claimed that the sample is representative of China’s health care. However, we deliberately developed measures of individual-level resources (professional efficacy, development opportunities and remuneration) and team-level resources (team cooperation, team learning, and team effectiveness) that are largely context-free, i.e., it seems plausible that these resources are salient across a wide range or occupations and cultures. If correct, this suggests that our findings may well be relevant (and may be generalized) across occupations and cultures. Furthermore, these context-generalized measures of individual-level resources and team-level resources tend to capture more of a stable resource characteristic than situation-specific resources (such as work shift and medical training). This strategy may facilitate the direct applicability of our measures to other contexts, such as teachers with high-education or blue-collar workers with low-education.

The findings presented here are basically in line with our expectations. However, it should be noted that in this study, the collaboration of team members refers to the interprofessional collaboration of doctors and nurses. This interprofessional collaboration is an essential component in any hospital. Doctors and nurses have different roles (and, possibly, different identities) in patient care. For example, doctors traditionally consider themselves as the dominant authority, while the main function of nursing is carrying out orders. This could mean that both individual crafting and collective crafting behaviors are linked with different features of the jobs of the participants. The intraprofessional collaboration in other job contexts (such as sale teams or teaching teams) may be different from the collaboration as it occurs between the hierarchically different jobs of doctors and nurses. In addition, different cultures foster different modes of cognitive processing (Kitayama, 2000). People in collectivist cultures (e.g., the Chinese participants in this study) are assumed to be relatively more sensitive to specific features of the interpersonal relationships they maintain and the social context in which they operate. These culture-dependent cognitive characteristics may influence people’s judgments about their own job crafting behaviors. In order to address this issue, in this study we used a broadly defined job crafting measure (i.e., the OJCS) to tap job crafting-centered values and in this sense we believe that the cultural context in which this study was conducted will not have biased our findings severely.

The third limitation of this study is that it used a cross-sectional design to capture the relationships among job-related resources, job crafting and work engagement. Such studies have limitations, on one hand, job-related resources and work engagement may be reciprocally related. People who are engaged in their work may be motivated to stay engaged by job crafting to increase the levels of job-related resources (Bakker and Demerouti, 2007, 2017); On the other hand, people craft their job by actively reconstructing and customizing it, a variety of job-related resources can be reorganized, restructured or reframed in the job crafting process. With a cross-sectional design, we cannot conclude that specific types of resources causally “lead to” employee well-being via job crafting. However, job-related resources (e.g., learning and developmental opportunities, remuneration and professional efficacy) do on average not exceed moderate levels, and are on average stable across longer periods of time (Sonnentag, 2017). Work engagement is a constantly changing and dynamic, rather than a lasting, state that is closely linked to task performance (Sonnentag, 2017). The notion that job crafting and job resources are dynamically and reciprocally related does not specify how job crafting and the relatively stable job-related resources affect each other, and neither does it tell us how the job-related resources affect work engagement. Our cross-sectional study captures relative stable of job-related resources, as well as more volatile psychological energy that is linked with momentary experiences in the work environment (Tuckey et al., 2018). In this sense, although with limitations, a cross-sectional study helps in building an integrated perspective on the relationships among job-related resources, job crafting and work engagement. Note that the relative stability of the job-related resources in this study has merely been assumed; it should be *demonstrated* in experimental or longitudinal designs, controlling the variation in specific resources which is typical for work engagement. Similarly, in this study work engagement is assumed to be a momentary work-related experience. However, we did not distinguish between state work engagement and trait work engagement. In future research, a diary study may uncover the relationships between relatively stable job-related resources and the dynamic component of work engagement.

## Conclusion

The current study shows that resources at both the individual and the team levels may promote individual work engagement through individual and collective job crafting, respectively. This finding provides a valuable contribution for understanding the effects of resources at different levels, as assumed by COR theory. This study adds to the COR literature by not only showing that resource-related processes operate at different levels, but also by exploring how individual work engagement can be promoted by multilevel job crafting.

## Ethics Statement

This study was conducted in accordance with the ethical guidelines set by the Institutional Review Board of Zhejiang University of Technology in China, with written informed consent from all subjects. Employees participated in our study voluntarily. The protocol was approved by the ZJUT Secretariat of Academic Committee of Zhejiang University of Technology, China. The permit number is 2017001.

## Author Contributions

QH led the work carried out on the study, including its conceptualization, research design, data collection and data analysis, and took the lead in writing the manuscript. WS and TT contributed to the conceptualization of this study and contributed to its write-up. AS was involved in the original conceptualization of the work. MD was involved in the statistical analyses of the data and contributed to its write-up.

## Conflict of Interest Statement

The authors declare that the research was conducted in the absence of any commercial or financial relationships that could be construed as a potential conflict of interest.
